# Developing a coding taxonomy to analyze dental regulatory complaints

**DOI:** 10.1186/s12913-020-05943-7

**Published:** 2020-11-25

**Authors:** Monika Roerig, Julie Farmer, Abdulrahman Ghoneim, Noha Gomaa, Laura Dempster, Krystal Evans, Wanda La, Carlos Quiñonez

**Affiliations:** 1 Dental Public Health, Faculty of Dentistry, University of Toronto, Toronto, Canada; 2 Oral Medicine, Schulich School of Medicine and Dentistry, University of Western Ontario, London, Canada; 3 Royal College of Dental Surgeons of Ontario, Toronto, Canada

**Keywords:** Complaint, Quality of Care, Regulation

## Abstract

**Background:**

As part of their mandate to protect the public, dental regulatory authorities (DRA) in Canada are responsible for investigating complaints made by members of the public. To gain an understanding of the nature of and trends in complaints made to the Royal College of Dental Surgeons of Ontario (RCDSO), Canada’s largest DRA, a coding taxonomy was developed for systematic analysis of complaints.

**Methods:**

The taxonomy was developed through a two-pronged approach. First, the research team searched for existing complaints frameworks and integrated data from a variety of sources to ensure applicability to the dental context in terms of the generated items/complaint codes in the taxonomy. Second, an anonymized sample of complaint letters made by the public to the RCDSO (*n* = 174) were used to refine the taxonomy. This sample was further used to assess the feasibility of use in a larger content analysis of complaints. Inter-coder reliability was also assessed using a separate sample of letters (*n* = 110).

**Results:**

The resulting taxonomy comprised three domains (Clinical Care and Treatment, Management and Access, and Relationships and Conduct), with seven categories, 23 sub-categories, and over 100 complaint codes. Pilot testing for the feasibility and applicability of the taxonomy’s use for a systematic analysis of complaints proved successful.

**Conclusions:**

The resulting coding taxonomy allows for reliable documentation and interpretation of complaints made to a DRA in Canada and potentially other jurisdictions, such that the nature of and trends in complaints can be identified, monitored and used in quality assurance and improvement.

**Supplementary Information:**

The online version contains supplementary material available at 10.1186/s12913-020-05943-7.

## Background

Complaints in healthcare settings are emotive, spontaneous, subjective, and complex, representing an expression of grievance or dispute that describes a service failure or unmet expectation [1–4]. A complaint is often made to seek a response from the accused healthcare provider and/or setting and to reach a resolution, whether it means securing an apology, an investigation and disciplinary action, or changes in practice to avoid future wrongdoing [5]. Complaints themselves are important, as they provide a wealth of information to inform service improvement. For example, through complaints, patients and the public provide an independent assessment of healthcare providers, organizations and systems grounded in the norms and expectations of society [6].

Considering a complainant’s perspective is especially important in gaining insight about patient and/or public expectations, especially in areas in service provision that may require improvement. Patients and the public evaluate service quality based on a broad spectrum of factors relating to their care, such as clinical components, the interpersonal skills of providers, the cost of services, and the physical environment of healthcare settings [7, 8]. Safety issues may also be highlighted in complaints, like clinical mistakes made by a healthcare professional (active failures) and factors that contribute to failures, including  policies, procedures and training (latent failures). For quality assurance and public protection, it is important to consider the ways in which negative experiences occur (or may occur) in order to develop new, enhanced and responsive solutions.

Analyses of healthcare complaints are primarily concerned with patient dissatisfaction and safety incidents. Methods include reviewing complaint letters or files, malpractice insurance claims, data from incident reporting systems, patient records, and/or conducting surveys [9–11]. A review by Hiivala et al. [9] of the dental literature regarding detectable safety incidents revealed that issues from various countries and dental disciplines share similar themes [9]. These themes relate to: treatment (e.g., errors, complications and poor skill); diagnostic and clinical assessments (e.g., faulty diagnosis, incomplete radiographic assessment); medications (e.g., adverse drug events); practice processes (e.g., infection control, documentation); consent and confidentiality; practitioner behaviour; and the health of the practitioner. This thematic grouping mirrors patient and public perceptions about the quality of care; that is, quality is influenced by factors inclusive and exclusive of clinical abilities.

The complexity of complaints and potential variability across service settings within dentistry can challenge the reliable extraction of narratives from patients and the public. Further, standardized analysis techniques are often lacking or unclear, and there is inconsistency in the selection of data sources and samples for these analyses [4]. For example, a study focus might be centred around the chief complaint or the most severe safety issue, and could include only complaints made by patients, or review of an entire case file. From a scientific and policy perspective, existing research in this area tends to demonstrate limited details about how analytical decisions were made, the number and types of researchers involved in the analytical process, and how reliability in data collection, analysis and reporting was assessed and established.

A standardized method for collecting, aggregating and analyzing complaints has been suggested to allow for reliable documentation and interpretation of complaints, such that trends can be accurately identified [1, 2]. For example, Reader and colleagues (2014) developed a now widely used complaint taxonomy based on a systematic review of patient complaints in healthcare systems. Their complaint taxonomy classifies complaints into clinical, management or relational issues and are represented as domains. Categories and sub-categories within the domain further characterize the complaint [1]. The taxonomy has been applied in various settings, including dentistry [12]. However, this taxonomy presents limitations specific to the dental context, as some issues are not applicable and dentistry-specific issues are missing [12]. As well, the contrasting structures and processes of healthcare and dental care systems may contribute to differences in perceived quality and safety [13].

The goal of this study was to develop a taxonomy for use in the reliable documentation and interpretation of complaints made to the Royal College of Dental Surgeons of Ontario (RCDSO), Canada’s largest dental regulatory authority (DRA), so that the nature of and trends in complaints can be identified, monitored and used in quality assurance and improvement.

## Method

### Context and approach

Every year, the RCDSO receives hundreds of complaints regarding dentists and/or dental care from patients, dental office staff, insurance companies, government agencies, other dental professionals, and any other member of the public. As part of its mandate to act in the public interest, the RCDSO investigates complaints to determine appropriate outcomes focused on public protection. In addition to the statutory requirement to respond and investigate all complaints, the DRA was interested in better understanding issues faced in the dental care context to devise effective interventions that minimize their occurrence. As such, they engaged our research team (CQ, LD, MR, JF, AG, NG) to conduct a review of complaints. To start the research process, it was determined that a coding taxonomy, or framework, was necessary to understand, count, and synthesize complaints. The research team sought and received approval for scientific merit from the Faculty of Dentistry, University of Toronto and ethical approval from the Office of Research Ethics at the University of Toronto (REB#35975).

### Purpose of the taxonomy

The purpose for developing the coding taxonomy was to systematically describe complaints made by the public to a DRA regarding dental care and dentists. The taxonomy would be used to perform a systematic and robust analysis of the content of written complaints made by the public to the RCDSO. Content analysis is defined as a systematic, replicable technique for organizing and tabulating text achieved through the process of ‘coding,’ where a given unit of analysis is categorized as a ‘code’ and represented as quantitative data [14, 15]. In a content analysis, the taxonomy provides a mechanism by which to systematically code and interpret textual material.

### Description of data sources

The research team received a random sample of 2199 letters of complaint (LOC) from the RCDSO. These LOCs were the initial letter received from a complainant to the DRA. The LOC were anonymized by the RCDSO prior to transfer to the research team, leaving only the initials of the complainant, provider(s) and staff member(s) being described in the LOC. This ensured accurate interpretation of the material within the LOC, while making sure that all information was deidentified and anonymous. LOC were either scanned or originally received by the RCDSO in an electronic format, and this material was transferred to the research team on encrypted USB drives (IronKey™ D300). A total of 174 LOC from the original sample were used to develop and pilot test the taxonomy, including letters from 2007 (*n* = 14), 2008 (*n* = 30), 2009 (*n* = 30), and 2016 (*n* = 100). An additional 110 LOC from the original sample were used to assess inter-coder reliability of the final taxonomy.

### Development of the dental complaint taxonomy

The taxonomy was developed through a series of methods, including a literature review and qualitative review of sample of LOCs. First, the research team began by constructing a conceptual framework of potential complaints in dental care by reviewing existing frameworks and taxonomies relevant to quality of care, access to care, and patient complaints in healthcare settings [1, 16, 17]. The team also carried out a literature search to identify articles related to patient satisfaction, clinical malpractice and complaints specific to dental care to further anticipate potential complaint issues in dentistry. Table 1 describes prominent concerns of discontent and dissatisfaction identified from this review. All of the above complaint issues were then merged and adapted to reflect potential complaints in the dental care setting.

**Table 1:** Dental complaint issues identified in the literature

Theme	Complaint issue
Examination and diagnosis	Incomplete or improper patient examination ^1–4^ Diagnostic errors, including missed diagnosis and misdiagnosis ^2–11^ Failing or refusing to refer the patient to another dental professional ^1, 2, 10, 12–14^
Treatment	Performing an inappropriate, unnecessary or inadequate procedure ^2, 6–8, 12, 13, 15–17^ Failed, delayed or incomplete treatment ^9, 12, 14, 18^ Procedural errors, including performing the procedure on the incorrect tooth or site, inhaling or swallowing an object, choking or brief respiratory arrest, file fractures, improper filling, adverse reaction to latex or materials, soft tissue burns from heated instruments, and perforation; ^1, 3, 4,7–9, 11–13, 19–22, 24^ Adverse drug or anaesthesia reaction ^5, 12, 22, 25^ including overdose ^1, 8, 20^ Treatment complications or iatrogenic injuries, including infections, soft tissue injuries, persistent bleeding, injuries to adjacent tooth, nerve damage or injury, eye damage, and damaged, broken, or tooth loss; ^4, 7, 8,9, 11, 12,19, 20,21, 26^ Unmet treatment expectations or dissatisfaction, negligence and emotional distress ^2, 5, 8, 14, 18, 27,28^ Inappropriate hygiene and infection control ^1, 2, 4–6, 22, 27^ Equipment failure ^12, 20, 22^ The experience of pain or poor pain management 7,^12, 19, 29, 30^
Practice processes	Clerical errors and problems with recordkeeping ^1–3, 6, 12, 13, 20–22, 24, 28^ Procedure fees, service cost and billing ^2, 5, 14, 15, 18, 27, 30, 31^
Interpersonal skills and professionalism	Poor communication, information sharing and unprofessional behaviour ^2, 3, 5,6, 7, 13, 17, 19, 21, 24, 27,28, 29, 30, 31^ Lack of shared decision making or autonomy^18, 21^ Loss of trust ^15, 28, 29^ Substance use or incapacity ^1, 28^ Breach of confidentiality or privacy ^5, 6, 18, 21^ Lack of informed consent ^2,4,6,7, 10, 13, 16, 17, 21, 26, 27^

^1^Hiivala et al. 2016; ^2^Postma et al. 2011; ^3^Ashkenazi et al. 2011; ^4^Milgrom 1985; ^5^Fredericks-Younger, Handelman-Yellin, and York 2017; ^6^Brown 2015; ^7^Gulati et al. 2012; ^8^Obadan, Ramoni, and Kalenderian 2015; ^9^Cronström, Öwall, and René 1998; ^10^Milgrom et al. 1994; ^11^Perea-Pérez et al. 2014; ^12^Hashemipour et al. 2013; ^14^Modolo, Calvielli, and Antunes 1999; ^15^Riley et al. 2012; ^16^ Ozdemir et al. 2005; ^17^Singh, Mizrahi, and Korb 2009; ^18^Lok, Kruger, and Tennant 2007; ^19^Bjørndal and Reit 2008; ^20^Hiivala, Mussalo-Rauhamaa, and Murtomaa 2013; ^21^Marei 2013; ^22^Thusu, Panesar, and Bedi 2012; ^23^Bilder, Hazan-Molina, and Aizenbud 2011; ^24^Pinchi et al. 2013; ^25^Chicka et al. 2012; ^26^Perea-Pérez et al. 2011; ^27^Hopcraft and Sanduja 2004; ^28^Hiivala, Mussalo-Rauhamaa, and Murtomaa 2014; ^29^Krause, Bremerich, and Rustemeyer 2001; ^30^Calnan, Dickinson, and Manley 1999; ^31^Sachdeo et al. 2012

Second, one research team (MR) open coded a subsample of LOC, resulting in additions to the existing list of complaint codes in the initial framework. These complaint codes were grouped by theme, compared to the initial taxonomy, and integrated into an initial taxonomy. This process allowed the team to (i) assess the content validity of the complaint codes and categories from the literature; (ii) capture any additional codes reflective of the diverse sample of complaints; and (iii) develop initial code definitions.

The taxonomy underwent three iterations of testing and revision. To begin this testing, three team members (MR, LD, CQ) coded the same randomly selected LOC (*n* = 5) from the 2016 subsample. A step-by-step coding method was used whereby the: LOC was read in its entirety before coding; complainant’s perspective was always taken as primary when coding; and codes in an Excel spreadsheet that contained all of the usable codes from the taxonomy were checked off. Any new codes recommended by the three team members and associated code definitions were added and/or other codes collapsed based on consensus among the three team members.

### Pilot testing and refinement

The research team (MR, LD, CQ) conducted a pilot test of the coding process and content of the initial taxonomy by using a random subsample of LOC from 2016 (*n* = 100). Then, three additional team members (JF, AG, NG) were trained to code complaints as per the step-by-step coding method described above in a separate sample of LOCs (*n* = 14). This provided another opportunity to refine and include new complaint codes and code definitions.

### Quality of the complaint coding and taxonomy development process

The research team used three main steps to ensure quality in the complaint coding and taxonomy development process. First, team members independently coded LOC throughout the taxonomy development phase to ensure that codes identified within LOC could be confirmed by other researchers (confirmability) [18]. Second, multiple coders and consensus meetings served as a form of investigator triangulation to ensure that code complaints were interpreted consistently (credibility). Finally, recoding the same complaints helped assess and ensure stability of coding over time (dependability).

### Inter-coder reliability assessment

Inter-coder reliability was qualitatively assessed between all six coders (MR, JF, AG, NG, LD, CQ) using a sub-sample of LOC from 2007 (*n* = 14). Each member independently coded the same letters using the step-by-step coding method, submitted their results for tabulation, and met to discuss discrepancies. Using the same approach, a separate random sample of LOC (*n* = 110) from all years (2007–2017) was used to quantitatively assess inter-coder reliability at the problem category level in the taxonomy. For this sample, Krippendorf’s alpha (α), which estimates the level of agreement in coding among multiple coders, was calculated through SPSS® statistical software using the kalpha macro [14, 19].

### Supplemental information to the coding taxonomy

In order to systematically describe the complaint’s features, members of the research team (MR, CQ, and LR) also developed a separate coding scheme. This scheme included information on the following: i) the complainant source (patient, family member, dentist, third party/other); ii) the number and type of provider(s) and/or clinical staff involved in the complaint; and iii) what (if any) clinical area(s) the complaint referred to. Again, the step-by-step coding method ensured that coders read the complaint once in its entirety before assigning codes and before completing a complaint description form where the above information was noted.

## Results

**Fig. 1:**
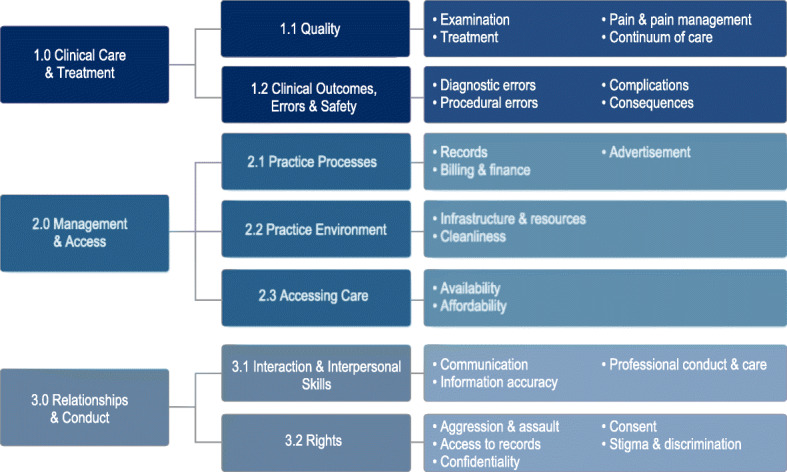
Overview of complaint taxonomy

The final taxonomy covers three domains (Clinical Care and Treatment; Management and Access; Relationships and Conduct), seven problem categories (Quality; Clinical Outcomes, Errors and Safety; Practice Processes; Practice Environment; Accessing Care; Interaction and Interpersonal Skills; Rights), 23 subcategories, and 106 complaint codes (Fig. 1).

### Domain 1: clinical care and treatment

The Clinical Care and Treatment (1.0) domain (Table 2) pertains to issues relating to the quality and safety of dental services, including two main problem categories: (i) Quality (1.1) defined as inadequate, inappropriate or unreliable service; and (ii) Clinical Outcomes, Errors and Safety (1.2) defined as clinical errors, incidents and outcomes.

**Table 2:** Complaint taxonomy, Domain 1: Clinical care and treatment

Problem category	Problem sub-category	Complaint codes	
1.1 Quality	1.1.1 Examination	• Incomplete examination • No action or examination	• Performed unnecessary or incorrect test/examination
	1.1.2 Treatment	• Failed treatment • Incomplete treatment • No action or treatment • Recommended unnecessary dental service	• Performed unnecessary or incorrect dental service • Performed dental service outside abilities • Supervised neglect • Unmet expectations/Dissatisfied
	1.1.3 Pain and pain management	• Pain • Poor acknowledgement of patient’s pain	• Provider would not prescribe for pain
	1.1.4 Continuum of care	• Miscommunication between practitioners • Failed to consult or make referral	
1.2 Clinical Outcomes, Errors and Safety	1.2.1 Diagnostic errors	• Incorrect diagnosis • Missed diagnosis	• Laboratory or imaging error
	1.2.2 Procedural errors	• Anaesthesia error • File fracture • Inhalation or ingestion of object	• Procedure on wrong tooth or site • Procedure technique incorrect • *Other procedural errors*
	1.2.3 Complications	• Adverse reaction to dental materials • Adverse reaction to drugs • Damaged or broken tooth • Excessive bleeding • Excessive swelling • Headache or migraine	• Infection • Nerve injury • Malocclusion • Trauma to lips, tongue, inside mouth • *Other complications or iatrogenic injuries*
	1.2.4 Consequences of clinical error	• Additional fees for subsequent procedure • Dental anxiety or fear	• Impacted quality of life
		• Consequence resulted in patient seeking alternative care from: - Another dental professional - Primary care physician - Medical specialist - Hospital and emergency services - Allied health or complimentary and alternative medicine (CAM) professional	

Quality (1.1) problem sub-categories include issues relating to examination (or diagnostic services), dental treatments, pain and pain management, and the continuum of care. Sub-categories of the Clinical Outcomes, Errors and Safety (1.2) category include diagnostic and procedural errors, complications, and consequences of clinical errors.

### Domain 2: management and access

The Management and Access (2.0) domain (Table 3) pertains to issues relating to the environment and clinic within which services were provided. The three problem categories are: (i) Practice Processes (2.1), defined as the processes of recordkeeping, billing and advertisement; (ii) Practice Environment (2.2), defined as the physical characteristics and resources of the clinic and resources; and (iii) Accessing Care (2.3), defined as the ability of persons to access clinic staff and clinical services.

**Table 3:** Complaint taxonomy, Domain 2: Management and access

Problem category	Problem sub-category	Complaint codes	
2.1 Practice Processes	2.1.1 Records	• Inadequate documentation • Incorrect documentation	• Falsified
	2.1.2 Billing and finances	• Billing irregularity • Insurance misuse • Not given receipt	• Payment harassment • Third-party creditor
	2.1.3 Advertisement	• False or misleading advertising • Other inappropriate advertising	
2.2 Practice Environment	2.2.1 Infrastructure and resources	• Lack of accommodation for disability • Lack of resources	
	2.2.2 Cleanliness	• Dirty environment • Unsanitary environment or equipment	
2.3 Accessing Care	2.3.1 Availability	• Delay or problems with scheduling • Long clinic wait time • Dropped patient	• Failed to reply to patient inquiry • Would not accept as patient • Lack of emergency care contact or resource
	2.3.2 Affordability	• Excessive charges or unreasonable fees • Unaffordable or expensive	• Inadequate information regarding procedure fees

### Domain 3: relationships and conduct

The Relationships and Conduct (3.0) domain (Table 4) pertains to issues relating to the behaviour of providers or any member of a clinic’s staff towards the patient or complainant. The two problem categories include: (i) Interaction and Interpersonal Skills (3.1), defined as inadequate, inaccurate or unprofessional communication, sharing of information and conduct; and (ii) Rights (3.2), defined as the violation of rights by dental clinic or dental staff.

**Table 4:** Complaint taxonomy, Domain 3: Relationships and conduct

Problem category	Problem sub-category	Complaint codes	
3.1 Interaction and Interpersonal Skills	3.1.1 Communication	• Failed to adequately inform patient of condition or diagnosis • Failed to answer question	• Insufficient follow-up • Language barriers
	3.1.2 Information accuracy	• Incomplete or inadequate information • Conflicting or inconsistent information	• Inaccurate information
	3.1.3 Professional conduct and care	• Concern disregarded • Distrust • Lacks compassion or insensitive • Rude or disrespectful	• Lack of shared decision making • Rushed, inattentive or distracted • Outside scope of dentistry • Suspected substance abuse or incapacity
3.2 Rights	3.2.1 Infrastructure and resources	• Lack of accommodation for disability • Lack of resources	
	3.2.2 Access to Patient Records	• Challenges in retention, access and/or transfer of patient record	
	3.2.3 Confidentiality and privacy	• Breach of confidentiality • Violation of patient privacy	
	3.2.4 Consent	• Coerced or mislead • Failed to disclose treatment information and/or risks	• Performed treatment without appropriate consent
	3.2.5 Stigma and discrimination	• Discrimination based on: - Class/Income - Health Status - Gender - Political views	- Race - Religion/Belief - Sexual orientation - Other forms of stigma or discrimination

### Inter-rater reliability results

Overall, the average Krippendorf alpha (α) estimate for the problem categories was 0.763 (95% confidence interval ([CI]: 0.716–0.805) and sub-categories was α = 0.658 (95% CI: 0.423–0.820). Problem categories that reached substantial reliability were 1.2 Clinical Outcomes, Errors and Safety (α = 0.827, 95% CI: 0.812–0.844), 2.1 Practice Processes (α = 0.845, 95% CI: 0.808–0.876) and 2.2 Practice Environment (α = 0.855, 95% CI: 0.741–0.944). All estimates and 95% CIs at the problem category level were above 0.6, with the lowest reliability estimate reported in the 2.3 Accessing Care category (α = 0.665, 95% CI: 0.611–0.717). Detailed reliability results are available in a Supplemental file.

## Discussion

This paper describes the development of a dental complaint taxonomy to organize and quantify the issues contained within LOC made to Canada’s largest DRA. The process and resulting taxonomy can be used to determine areas of concern expressed by complainants, whom they complained about, and trends in these areas over time. While patient safety, quality of care, and patient satisfaction have been explored in the context of complaints regarding dental care, a comprehensive study identifying dental care concerns and their trends, expressed in formal complaints submitted to a DRA in Canada, has not previously been conducted.

While our process for developing a complaints taxonomy followed similar methods described by Reader and colleagues [1], it also incorporated additional methods (i.e., review of LOCs) and therefore the resulting taxonomy provides unique contributions to this field. For example, our taxonomy incorporates complaints unique to dentistry at the problem category and sub-category levels that are not captured in Reader’s taxonomy. This includes consideration for specific procedural errors, such as endodontic file fractures and dental-related complications (e.g. malocclusion, damaged tooth, etc.) that are absent or rare in other fields, yet can have significant impacts for patients. Also, compared to other dental complaint taxonomies and coding processes, our taxonomy demonstrates and reports reliability, which is important for ensuring that findings related to dental complaints can be reproduced [12, 20]. Importantly, other complaint coding approaches in dentistry have used codes that cover a broad range of complaints relevant to various dental and non-dental health disciplines, or taxonomies with a reduced number of domains and categories that are relevant to specific settings, such as undergraduate dental clinics [12, 20]. Thus, in order to ensure that our complaint taxonomy is suitable for all settings, future research could consider comparing the comprehensiveness and reliability of each of these dental complaint coding approaches.

Our taxonomy covers the same conceptual domains as Reader and colleagues, and includes problem categories and sub-categories that are consistent between both taxonomies [1]. Through our analysis of LOC, we populated similar categories and sub-categories as Reader and colleagues and other complaint taxonomies [2]. This suggests that there may be consistency in the type of complaints and concerns made by the public across dental and healthcare settings. One study that applied Reader’s taxonomy also identified consistency in the type of complaints reported across healthcare disciplines, yet also noted variation in the frequency of these complaints across disciplines [21]. Similarly, our findings suggest that codes within our taxonomy could be grouped at the domain level (e.g. clinical care and treatment, management and access) and compared across all health disciplines, whereas reporting at the problem category and subcategory level could be used to compare differences in complaints within and across dental disciplines.

There are various groups who could use and benefit from our dental complaint taxonomy, including DRAs, professional associations, educators, researchers, patients and the public. First, DRAs could use the taxonomy to monitor the type and frequency of complaints made by the public over time or across jurisdictions [22]; in other words, findings could be used for quality assurance and improvement activities. Second, as our taxonomy was developed through complaints made by the public, educators, associations and regulators could use the taxonomy and our findings to identify topics relevant for undergraduate and graduate dental education and for continuing dental education opportunities for dental professionals. For example, this could facilitate the development of learning modules and courses that focus on quality of care, patient-centred care, patient satisfaction, and/or reinforcing the importance of interpersonal relationships in clinical care. Third, future research may consider developing scores and attributing weights to codes within our taxonomy to describe the seriousness or severity of complaints [4]; this could be used by researchers and DRAs to assess the degree of harm in the context of patient safety; however more information about the complaint may be required. Finally, using the taxonomy in the above and other ways would ideally lead to patient and public benefit through improved and enhanced clinical care, and/or through enhancing transparency and accountability initiatives.

There were some limitations in our study that are worth discussing. First, our process was not able to capture rare and unique issues, such as informal interactions between a dentist and a complainant. Some LOCs were also difficult to interpret because they were vague in description. Second, we developed a dental complaint taxonomy by using LOC that served as the initial contact between the complainant and the DRA. Other relevant information is held within complaint files, such as interviews, patient charts, and complainant responses, which could uncover additional risks or patient safety issues that do not arise within the initial LOC. Nevertheless, our approach ensures that issues and problems relevant to the public, from the perspective of members of the public, are captured. Finally, our reliability results at the problem category level identified categories that could be difficult to assess consistently. Such a concern has also been raised by other researchers who acknowledge challenges with complaint taxonomies and patient safety incidents and suggest that multiple coders could ensure that all relevant codes are captured [21, 23]. Despite these limitations, our study suggests that, as presented, the dental complaint taxonomy can be used in a content analysis of complaints made to a DRA.

## Conclusion

While some degree of error-making is inherent in all healthcare settings, identifying and classifying complaints serves a pivotal role to understanding patient and public expectations of healthcare systems, settings, and providers. The dental complaint taxonomy described in this paper is comprehensive and has demonstrated good reliability among a range of coders. This suggests that the taxonomy is a suitable tool for systematically reviewing complaints, which can help regulators and other stakeholders respond to public needs and reduce the prevalence and severity of negative incidents and outcomes.

## Supplementary Information

**Additional file 1: Table 1.** Summary of inter-rater reliability values at the problem category level.

## Data Availability

As per the nature of the data (letter of complaints) used in this study, it and other associated materials are not publicly available due to privacy and confidentiality reasons and are unavailable upon request.

## Abbreviations

CI: Confidence interval
DRA: Dental Regulatory Authorities
LOC: Letters of Complaint
RCDSO: Royal College of Dental Surgeons of Ontario

## References

[1] Reader TW, Gillespie A, Roberts J (2014). Patient complaints in healthcare systems: a systematic review and coding taxonomy. BMJ Qual Saf.

[2] Montini T, Noble AA, Stelfox HT (2008). Content analysis of patient complaints. Int J Qual Health Care.

[3] Mulcahy L, Tritter JQ (1998). Pathways, pyramids and icebergs? Mapping the links between dissatisfaction and complaints. Sociol Health Illn.

[4] Gillespie A, Reader TW (2018). Patient-centered insights: using Health Care complaints to reveal hot spots and blind spots in quality and safety. Milbank Q.

[5] Lloyd-Bostock S, Mulcahy L (1994). The social psychology of making and responding to hospital complaints: an account model of complaint processes. Law Policy.

[6] Toop L (1998). Primary care: core values patient centred primary care. Bmj..

[7] Gerbert B, Bleecker T, Saub E (1994). Dentists and the patients who love them: professional and patient views of dentistry. J Am Dent Assoc 1939.

[8] Williams SJ, Calnan M (1991). Convergence and divergence: assessing criteria of consumer satisfaction across general practice, dental and hospital care settings. Soc Sci Med.

[9] Hiivala N, Mussalo-Rauhamaa H, Tefke H-L, Murtomaa H (2016). An analysis of dental patient safety incidents in a patient complaint and healthcare supervisory database in Finland. Acta Odontol Scand.

[10] Hiivala N, Mussalo-Rauhamaa H, Murtomaa H (2015). Can patients detect hazardous dental practice? A patient complaint study. Int J Health Care Qual Assur.

[11] Milgrom P, Fiset L, Whitney C, Conrad D, Cullen T, O’Hara D (1994). Malpractice claims during 1988-1992: a national survey of dentists. J Am Dent Assoc 1939.

[12] Fredericks-Younger JM, Handelman-Yellin ML, York JA (2017). Developing a relevant taxonomy to assess dental school clinic patient complaints. J Dent Educ.

[13] Yamalik N, Perea PB (2012). Patient safety and dentistry: what do we need to know? Fundamentals of patient safety, the safety culture and implementation of patient safety measures in dental practice. Int Dent J.

[14] Krippendorff K. Content analysis: an introduction to its methodology. Thousand Oaks: Sage Publications, Inc; 2018.

[15] Stemler S (2000). An overview of content analysis. Pract Assess Res Eval.

[16] Campbell SM, Roland MO, Buetow SA (2000). Defining quality of care. Soc Sci Med.

[17] Penchansky R, Thomas JW. The concept of access: definition and relationship to consumer satisfaction. Med Care. 1981:127–40. 10.1097/00005650-198102000-00001 7206846

[18] Korstjens I, Moser A (2018). Series: Practical guidance to qualitative research. Part 4: Trustworthiness and publishing. Eur J Gen Pract.

[19] Hayes AF, Krippendorff K (2007). Answering the Call for a Standard Reliability Measure for Coding Data. Commun Methods Meas.

[20] Data on Written Complaints in the NHS. NHS Digital.. Available from: https://digital.nhs.uk/data-and-information/publications/statistical/data-on-written-complaints-in-the-nhs [cited 2020 Apr 9].

[21] Harrison R, Walton M, Healy J, Smith-Merry J, Hobbs C (2016). Patient complaints about hospital services: applying a complaint taxonomy to analyse and respond to complaints. Int J Qual Health Care.

[22] van Dael J, Reader TW, Gillespie A, Neves AL, Darzi A, Mayer EK. Learning from complaints in healthcare: a realist review of academic literature, policy evidence and front-line insights. BMJ Qual Saf. 2020;bmjqs-2019-009704. [cited 2020 May 21]. 10.1136/bmjqs-2019-009704. 10.1136/bmjqs-2019-009704 PMC7398301 32019824

[23] Taib IA, McIntosh AS, Caponecchia C, Baysari MT (2011). A review of medical error taxonomies: A human factors perspective. Saf Sci.

