# Supplementary material for: Developing a coding taxonomy to analyze dental regulatory complaints
Source: BMC Health Serv Res. 2020 Nov 25;20:1083. doi: 10.1186/s12913-020-05943-7 (PMC7691083; doi:10.1186/s12913-020-05943-7)
Supplement: Supplementary file 1 — Additional file 1: Table 1. Summary of inter-rater reliability values at the problem category level. [file 12913_2020_5943_MOESM1_ESM.docx]

**Supplemental file**

Appendix Table 1. Summary of inter-rater reliability values at the problem category level.

| Domain | Problem categories | Krippendorff’s α [95% CI] |
| --- | --- | --- |
| 1.0 Clinical care and treatment | 1.1 Quality | 0.718 [0.689, 0.743] |
|  | 1.2 Clinical Outcomes, Errors, and Safety | 0.827 [0.812, 0.844] |
| 2.0 Management and access | 2.1 Practice Processes | 0.845 [0.808, 0.876] |
|  | 2.2 Practice Environment | 0.855 [0.741, 0.944] |
|  | 2.3 Accessing care | 0.665 [0.611, 0.717] |
| 3.0 Relationship and conduct | 3.1 Interaction and Interpersonal Skills | 0.692 [0.666, 0.720] |
|  | 3.2 Rights | 0.741 [0.686, 0.788] |
